# Non-alignment stagnation-point flow of a nanofluid past a permeable stretching/shrinking sheet: Buongiorno’s model

**DOI:** 10.1038/srep14640

**Published:** 2015-10-06

**Authors:** Rohana Abdul Hamid, Roslinda Nazar, Ioan Pop

**Affiliations:** 1 Institute of Engineering Mathematics, Universiti Malaysia Perlis, Perlis, Malaysia; 2 School of Mathematical Sciences, Faculty of Science & Technology, Universiti Kebangsaan Malaysia, Selangor, Malaysia; 3 Department of Mathematics, Babeş-Bolyai University, Cluj-Napoca, Romania

## Abstract

The paper deals with a stagnation-point boundary layer flow towards a permeable stretching/shrinking sheet in a nanofluid where the flow and the sheet are not aligned. We used the Buongiorno model that is based on the Brownian diffusion and thermophoresis to describe the nanofluid in this problem. The main purpose of the present paper is to examine whether the non-alignment function has the effect on the problem considered when the fluid suction and injection are imposed. It is interesting to note that the non-alignment function can ruin the symmetry of the flows and prominent in the shrinking sheet. The fluid suction will reduce the impact of the non-alignment function of the stagnation flow and the stretching/shrinking sheet but at the same time increasing the velocity profiles and the shear stress at the surface. Furthermore, the effects of the pertinent parameters such as the Brownian motion, thermophoresis, Lewis number and the suction/injection on the flow and heat transfer characteristics are also taken into consideration. The numerical results are shown in the tables and the figures. It is worth mentioning that dual solutions are found to exist for the shrinking sheet.

Stagnation-point flows are a fundamental aspect of fluid mechanics. The solution for two-dimensional stagnation-point flow was given by Hiemenz^1^, while that for axisymmetric stagnation-point flow was given by Homann^2^ (see Bejan^3^). In stagnation point flow, a rigid wall or a stretching surface occupies the entire horizontal *x*-axis, the fluid domain is *y* > 0 and the flow impinges on the wall either orthogonal or at an arbitrary angle of incidence. This simple model of oblique stagnation point would enable us to understand how a boundary layer begins to develop and therefore, to determine its evolution from the stagnation point whose location is hence of great practical importance. It should be noticed that solutions do not exist for a shrinking sheet in an otherwise still fluid, since vorticity could not be confined in a boundary layer. However, with an added stagnation flow to contain the vorticity, similarity solutions may exist. These solutions are also exact solutions of the Navier–Stokes equations (see Wang^4^).

Nanofluid is a term first introduced by Stephen U.S. Choi in 1995 to describe the fluid that can enhance the heat transfer^5^. The main goal of nanofluid is to achieve the best thermal properties of a base fluid with a possible reduction of the volume of nanoparticles^6^. There are many studies that have been conducted to understand the process that occurs in nanofluid whether theoretical, numerical or experimental. In fact, many researchers have taken the initiative to make a review of studies that have been conducted. For example, Daungthongsuk and Wongwises^7^ have commented the research on forced convection heat transfer in nanofluid that has been done theoretically and experimentally. They found that the heat transfer coefficient in nanofluid is higher than normal base fluid and the heat transfer enhancement may be due to the following items; the use of nanoparticles to increase the thermal conductivity of the base fluid and chaotic motion of very small particles increases the turbulence in the fluid and thus expediting the process of energy exchange. Further, Lee *et al.*^8^ gave an overview on the thermal conductivity data, mechanisms and models in nanofluid by several research groups. They found that the inconsistency findings for each experimental thermal conductivity data is due to differences in sample quality, thermal conductivity dependence on many factors and differences in the measurement uncertainties. Therefore, they suggested the use of quality nanofluid, reference samples and equipment in a study. In addition, the issues involved in the mechanism to explain the thermal conductivity in nanofluid occur because of the lack of knowledge about the basic concepts of science to the mechanism. They also found that nanofluid model consisting of a combination of static and dynamic mechanism is seen to be more effective in describing the events in nanofluid. Nanofluid research is very important because of its use in various areas such as in the industrial cooling, electronics, electrical and many others. This field is not only carried out in the laboratory for academic purposes, but there are researchers who apply the nanofluid studies on the real devices to enhance the heat transfer performance of the devices^5^.

Formulations model for heat transfer by convection in the nanofluid have been proposed by many researchers. Among the famous is the model by Buongiorno^9^ which takes into account the Brownian motion and thermophoresis effect. Mathematical model introduced by Buongiorno were used in the studies by Nield and Kuznetsov^10^, Corcione *et al.*^11^, Tham *et al.*^12^ and recently by Garoosi *et al.*^13^. Generally, the findings of these studies found that the Brownian motion and thermophoresis parameters affect the boundary layer and the heat transfer in the nanofluid. Not only that, the Buongiorno model has been used to study the nanofluid past a stretching/shrinking sheet. Example of such study is the one by Rahman *et al.*^14^ that also considered the permeable surface of the sheet and with the second order slip velocity. They also employ a new boundary condition for the nanoparticles volume fraction at the surface of the shrinking sheet. Mustafa *et al.*^15^ also used the Buongiorno model to study the stagnation flow towards the stretching sheet using the homotopy analysis method (HAM). The research in the stretching or shrinking sheet is worth studying because it is crucial for the industrial applications such as the aerodynamic extrusion of plastic sheets, condensation process of metallic plate in a cooling bath and glass^16^, wire drawing and hot rolling^17^.

In this present study, we investigated the stagnation flow of nanofluid towards a permeable stretching/shrinking sheet where the flow and the sheet are not aligned. The non-alignment function is proposed by Wang^4^ and to the best of our knowledge, we are the first to consider the non-alignment function in the nanofluid that past a permeable stretching/shrinking sheet using Buongiorno’s model. According to Wang^4^, the non-alignment of the stagnation flow and the stretching/shrinking sheet can destroy the symmetry and complicates the flow field. The study by Wang^4^ has been extended by many researchers in various physical conditions such as a recent study by Najib *et al.*^18^. It should be mentioned that the governing system of ordinary differential equations are solved using the BVP4C function in Matlab. On the other hand, it is worth mentioning to this end that there are several other papers in the literature on symmetry breaking in flows or general analysis of flows^19^,^20^,^21^.

## Problem Formulation

**Figure 1:**
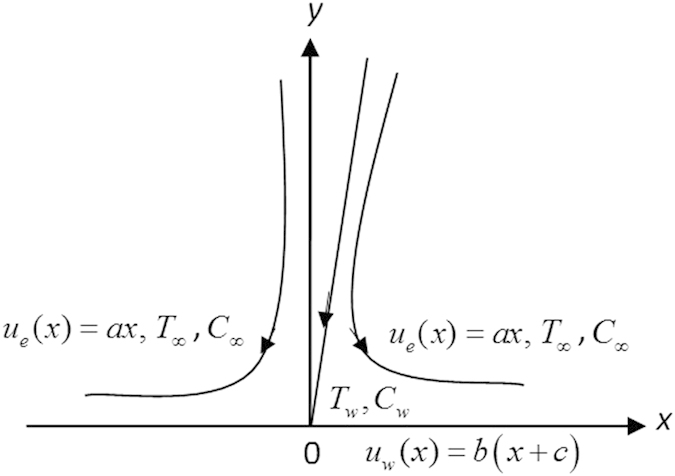
Physical model and coordinate system.

Consider the steady flow of a viscous nanofluid in the region *y* > 0 driven by a permeable stretching/shrinking surface located at *y* = 0 as shown in Fig. 1, where *x*, *y* and *z* are the Cartesian coordinates measured along the plate, normal to it and in the transversal direction, respectively. Let (*u*, *v*, *w*) be the velocity components in the directions *x*, *y* and *z*, respectively.

Following Wang^4^, we assume that for a two-dimensional stagnation point, the stretching/shrinking velocities of the surface are 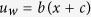 and *w* = *w*_0_, where *b* > 0 is the stretching rate (shrinking rate if *b* < 0), −*c* is the location of the stretching origin and *w*_0_ is the mass flux velocity with *w*_0_ < 0 for suction and *w*_0_ > 0 for injection or withdraw flow. It is also assumed that the fluid velocities outside the boundary layer (or the velocity of the inviscid nanofluid) are *u*_*e*_ = *ax* and *w*_*e*_ = −*az*, where *a* > 0 is the strength of the stagnation flow. Further, it is assumed that the uniform temperature and the uniform nanoparticle volume fraction of the plate are *T*_*w*_ and *C*_*w*_, respectively, while those of the ambient fluid are *T*_∞_ and *C*_∞_. It should be mentioned here that the stretching axis and the stagnation flow are not aligned^14^. Under the boundary layer approximations, the basic equations of the problem under consideration are, see Miklavčič and Wang^22^ and Kuznetsov and Nield^23^,


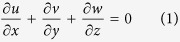



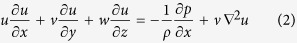



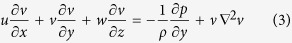



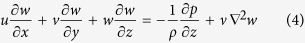



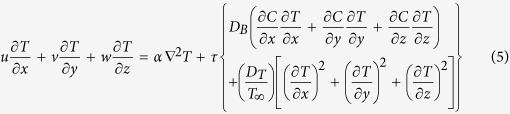



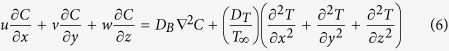


where *T* is the temperature of the nanofluid, *C* is the nanoparticle volume fraction, *p* is the pressure, *v* is the kinematic viscosity, *ρ* is the density of the nanofluid, *α* is the thermal diffusivity of the nanofluid, *D*_*B*_ is the Brownian diffusion coefficient, *D*_*T*_ is the thermophoretic diffusion coefficient and ∇^2^ is the Laplacean in the Cartesian coordinates *x*, *y* and *z*. Further, *τ* = (*ρc*)_*p*_/(*ρc*)_*f*_, where (*ρc*)_*f*_ is the heat capacity of the nanofluid and (*ρc*)_*p*_ is the effective heat capacity of the nanoparticle material. We assume that equations (1, 2, 3, 4, 5, 6) are subject to the boundary conditions


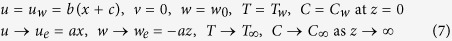


We look for a similarity solution of equations (1, 2, 3, 4, 5, 6) of the following form:


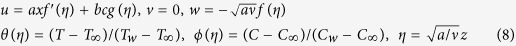


Substituting equation (8) into equations (2, 3, 4, 5, 6), we obtain the following ordinary differential equations:


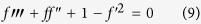



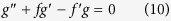



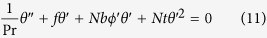



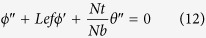


subject to the boundary conditions


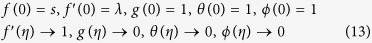


where 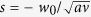 is the constant mass transfer parameter with *s* > 0 for suction and *s* < 0 for injection, respectively. It is worth also mentioning that *λ* = *b*/*a* is the stretching/shrinking parameter where *λ* > 0 corresponds to the stretching sheet and *λ* < 0 corresponds to the shrinking sheet. The dimensionless constants Pr, *Le*, *Nb* and *Nt* denote the Prandtl number, the Lewis number, the Brownian motion parameter and the thermophoresis parameter, respectively, which are defined as


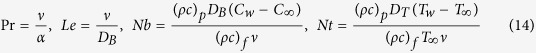


We notice that equations (9) and (10) have been studied by Wang^4^ for an impermeable sheet (*s* = 0) and in a viscous fluid.

The pressure *p* is given by


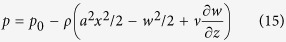


Quantities of physical interest in this problem are the skin friction coefficient *C*_*f*_, the local Nusselt number *Nu*_*x*_ and the Sherwood number *Sh*_*x*_ which are defined as


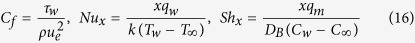


where *τ*_*w*_ is the skin friction or shear stress at the surface of the plate, *q*_*w*_ is the heat flux from the surface and *q*_*m*_ is the mass flux of the nanoparticle volume fraction from the surface, and are given by


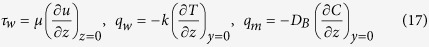


where *μ* is the dynamic viscosity of the fluid and *k* is the thermal conductivity of the nanofluid. Using equations (8), (16) and (17), we get


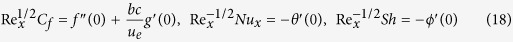


where Re_*x*_ = *u*_*e*_*x*/*v* is the local Reynolds number.

For two-dimensional flow, the dimensionless streamlines can be defined as


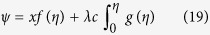


where 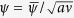 with 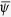 defined in the usual way as 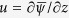 and 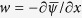.

## Results and Discussions

The system of ordinary differential equations (9, 10, 11, 12) with the boundary conditions (13) is solved numerically using the BVP4C function in Matlab. In this problem, solutions are obtained for the Prandtl number Pr = 6.8 (water-based nanofluid) and Lewis number *Le* = 2. The value of the suction parameter (*s* > 0) is chosen for 0.2 and the injection parameter (*s* < 0) is chosen for −0.2. Meanwhile, the stretching sheet (*λ* > 0) takes the value *λ* = 0.5 and the shrinking sheet (*λ* < 0) takes the value *λ* = −0.5. In order to verify the accuracy of the present method, comparison is made with Wang^4^ in the absence of the nanofluid parameters. The results are shown in Tables 1 and 2, and they are found to be in good agreement and thus give us confidence on the accuracy of the method.

Tables 3 and 4 show the numerical values for the reduced skin friction coefficient 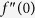, the reduced non-alignment function 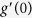, the reduced local Nusselt number 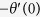 and the reduced nanoparticle volume fraction 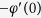 with the variations of the Brownian motion parameter *Nb*, the thermophoresis parameter *Nt* and the Lewis number *Le* for the stretching sheet and shrinking sheet, respectively. It is observed that variations of *Nb*, *Nt* and *Le* have no effect on 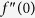 and 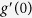. From the tables, it is found that the increase of the Brownian motion, thermophoresis and Lewis number parameters will reduce the local Nusselt number for both sheets, also for all suction/injection parameter *s* considered. Explanation of this phenomenon may be due to the enhancement of the collisions of the particles that results from the increasing of the Brownian motion and thermophoresis, which then increase the thermal boundary layer thickness, followed by the reduction of the local Nusselt number and the heat flux from the surface. Further, by increasing the Lewis number, the thickness of the thermal diffusion layer is becoming larger than the mass diffusion layer, which leads to lower values of 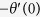. It should be noted that, increasing the Brownian motion, thermophoresis and Lewis number have different effects on the mass flux of the nanoparticle volume fraction. Generally, −*ϕ*′(0) is lower for the shrinking sheet when the fluid is injected.

**Figure 2:**
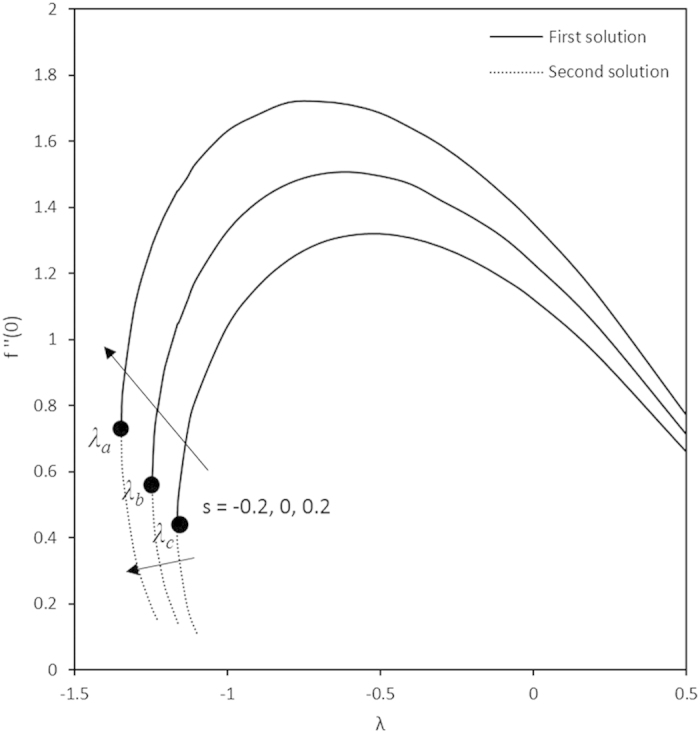
Variations of *f*′′(0) with different *λ.*

**Figure 3:**
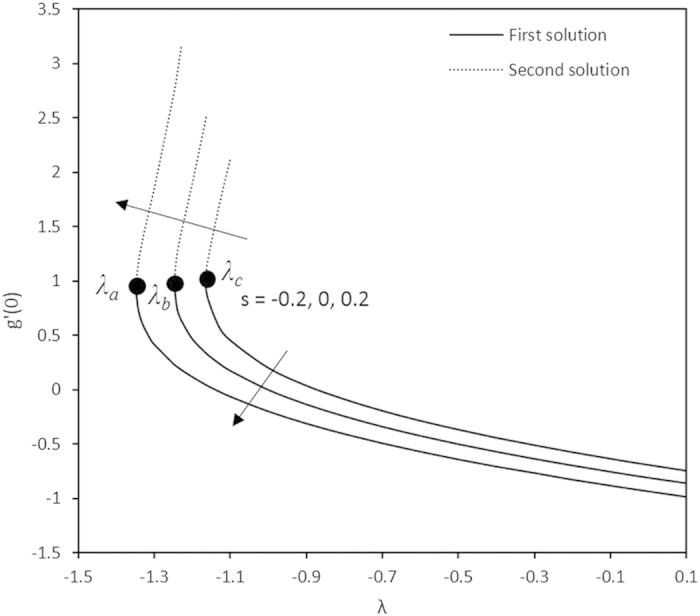
Variations of *g*′(0) with different values of *λ*.

**Figure 4:**
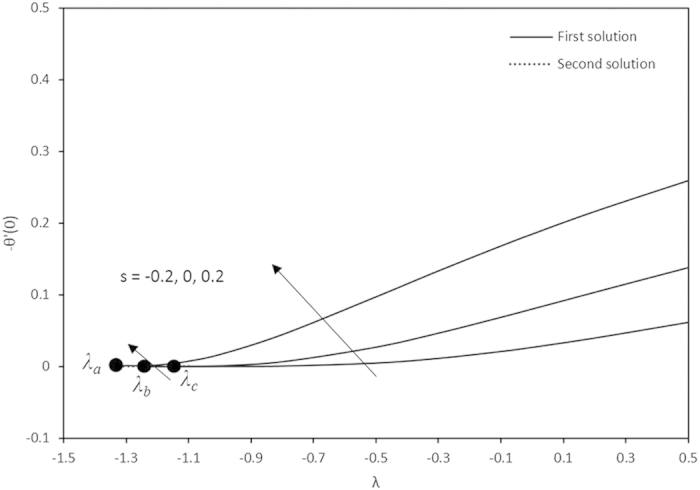
Variations of −*θ*′(0) with different values of *λ.*

**Figure 5:**
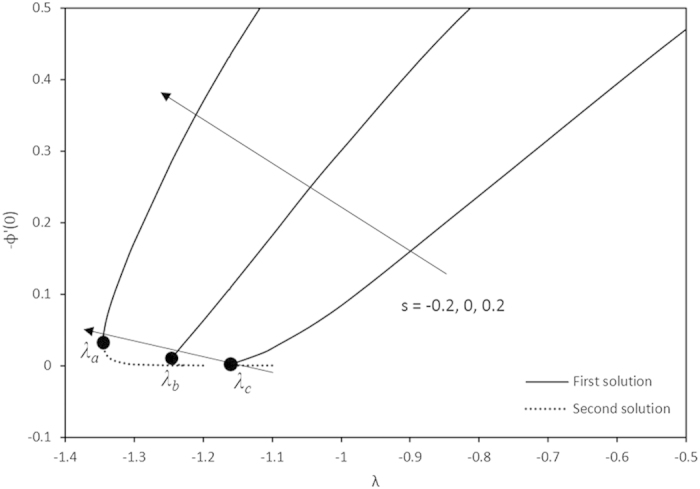
Variations of −*ϕ*′(0) with different values of *λ.*

**Figure 6:**
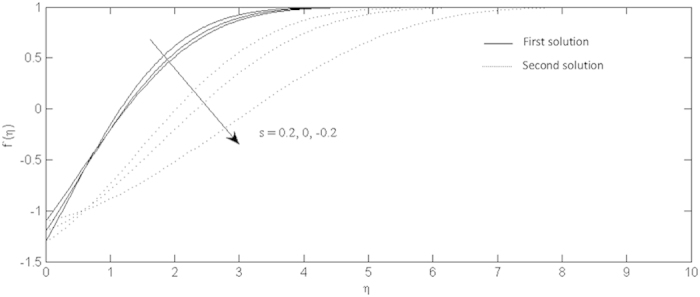
Effects of the suction and injection on the velocity profiles for shrinking sheet.

**Figure 7:**
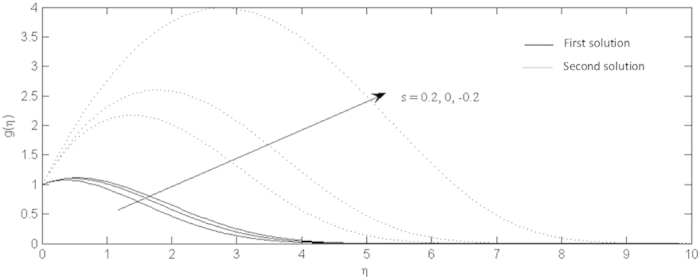
Effects of the suction and injection on the non-alignment function profiles for shrinking sheet.

**Figure 8:**
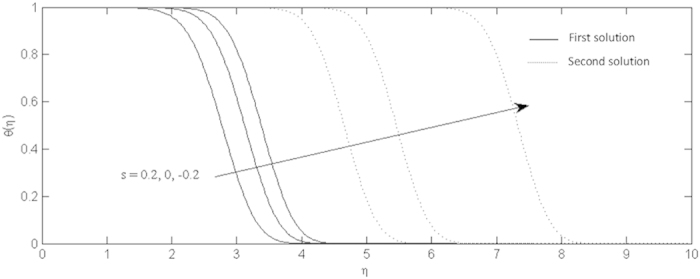
Effects of the suction and injection on the temperature profiles for the shrinking sheet.

**Figure 9:**
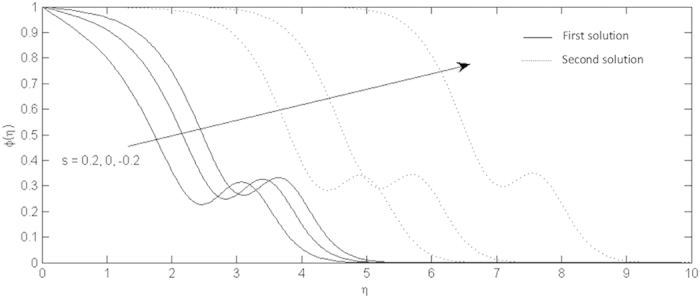
Effects of the suction and injection on the nanoparticle volume fraction profiles for the shrinking sheet.

On the other hand, the effects of the fluid suction and injection for the stretching and shrinking sheets are depicted on the Figs 2, 3, 4, 5, 6, 7, 8, 9, respectively. It is seen in the Figs 2 and 6 that imposition of the fluid suction tends to increase 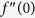 as well as the velocity profiles. Increment of 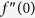 is not only because of the suction parameter but also due to the reduction of the value of *λ*. It is obvious that the interface shear stress is higher in the shrinking sheet. Moreover, we observed in the Figs 3 and 7 that the non-alignment function of the origin of the stagnation flow and the sheet can be reduced if the fluid is sucked into the surface of the sheet where it is more prominent in the shrinking sheet. It is worth mentioning that the non-alignment function is not affected by the variations in the Prandtl number. Further, Figs 4 and 8 show that the suction parameter also caused the reduction of the nanofluid temperature as the heat is transported into the interface of the sheet. Hence, as the thermal boundary thickness decreases, the heat flux from the surface is rising. The fluid suction also affected the nanoparticle volume fraction by reducing the profiles. This leads to the increasing of the interface mass transfer as shown in the Figs 5 and 9. On the contrary, the imposition of the fluid injection on the wall of the sheet produced the exact opposite behavior from the fluid suction.

**Figure 10:**
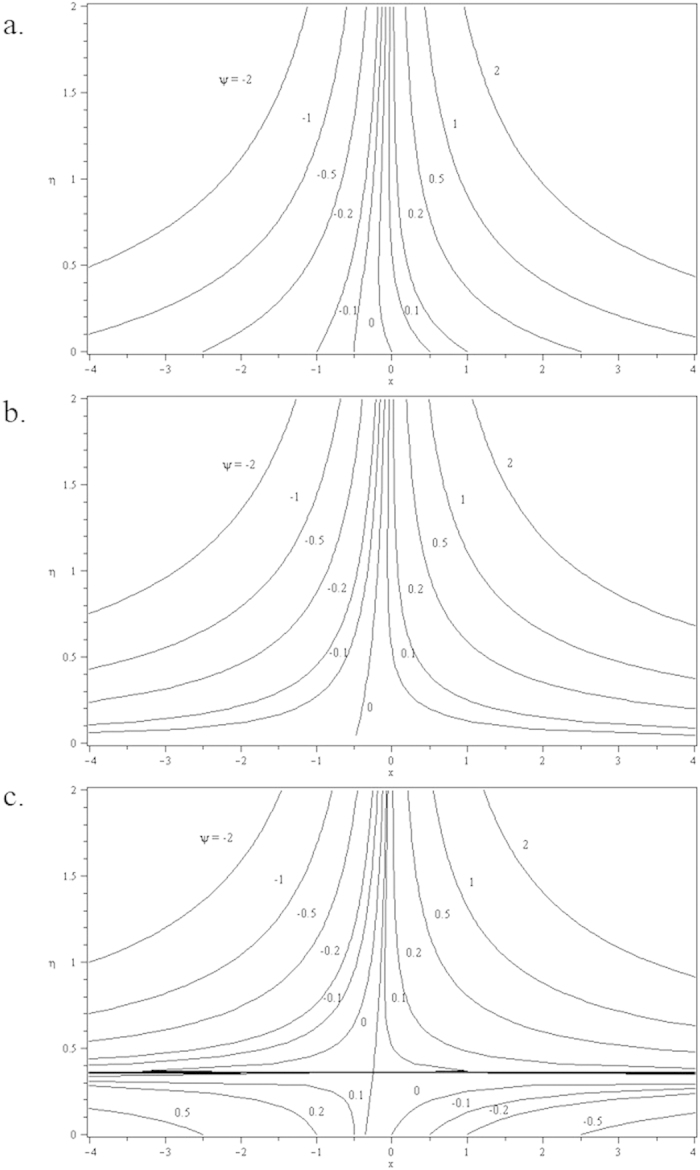
Streamlines for two-dimensional stretching sheet when *λ* = 0.5 and *c* = 0.5 for different values of *s*: (a) *s* = 0.2 (suction) (b) *s* = 0 (impermeable); (c) *s* = −0.2 (injection).

**Figure 11:**
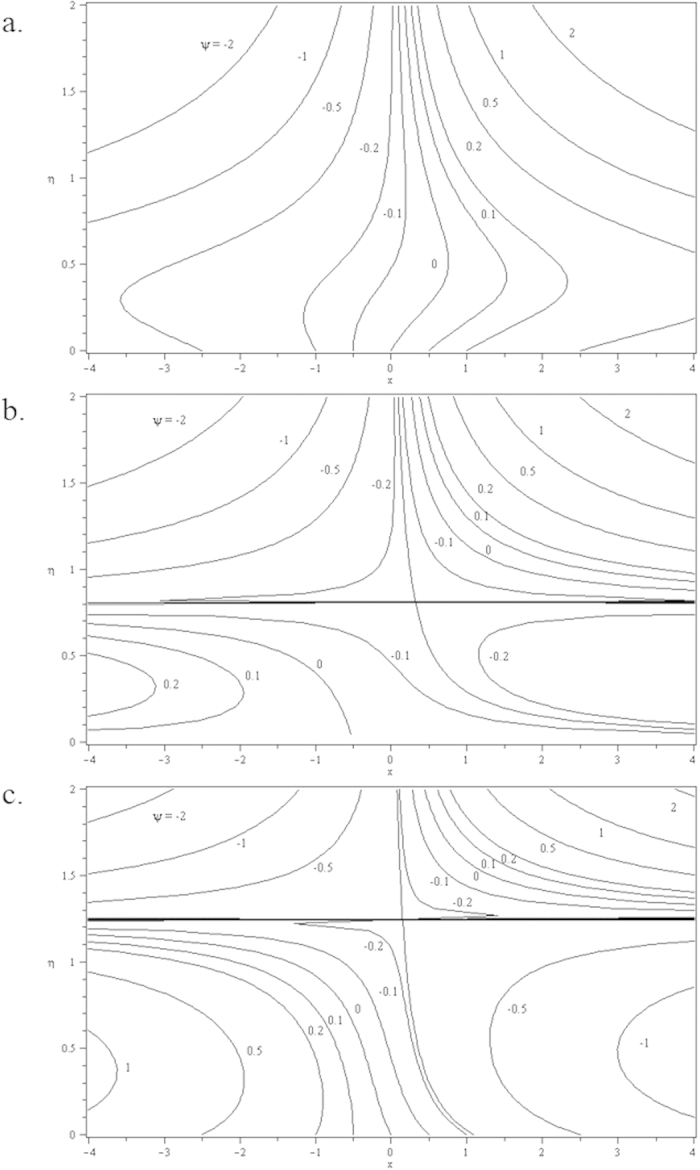
Streamlines for two-dimensional shrinking sheet when *λ* = −0.5 and *c* = 0.5 for different values of *s*: (a) *s* = 0.2 (suction) (b) *s* = 0 (impermeable); (c) *s* = −0.2 (injection).

Furthermore, in this problem the second solution is found to exist in the shrinking region where the critical values *λ*_*a*_ are for *s* = 0.2, *λ*_*b*_ for *s* = 0 and *λ*_*c*_ for *s* = −0.2 as reported in the Figs 2, 3, 4, 5. The values for the critical numbers are shown in the Table 5. It is observed that *λ*_*a*_ > *λ*_*b*_ > *λ*_*c*_. Not only that, for the second solution, the suction and injection parameters have the same effects as in the first solution. On the other hand, the streamlines for the present problem are shown in Figs 10 and 11 for the two-dimensional stretching and shrinking sheets, respectively. One can see that in both sheets, the suction parameter causes the flows being drag into the center. The fluid flows are also reduced. Meanwhile, when the fluid is injected through the surface, more flows are formed as shown in Figs 10(c) and 11(c).

## Conclusions

The present paper studied the steady laminar stagnation flow over a permeable stretching/shrinking sheet in the nanofluid using the Buongiorno’s model. It is found that the second solutions exist in the shrinking region. Further, the non-alignment function of the stagnation flow and the sheet complicates the flow fields and can be increased using the fluid injection. Moreover, the skin friction at the surface of the sheet is higher when the fluid is sucked into the surface. Different behavior is observed for the fluid injection. Generally, the Brownian motion, thermophoresis and Lewis number are the reducing factor of the heat transfer at the wall of both sheets. However, these parameters provide different effects for the rate of mass transfer. We mention to this end that the present paper can be extended by including the entropy effects into the governing equations by following, for example, the valuable books by Bejan^24^,^25^ and the papers by Bejan^26^, Adboud and Saouli^27^, Makinde^28^, Butt and Ali^29^,^30^, Rashidi *et al.*^31^, etc. and also by considering the constructal law^32^,^33^.

## Additional Information

**How to cite this article**: Hamid, R. A. *et al.* Non-alignment stagnation-point flow of a nanofluid past a permeable stretching/shrinking sheet: Buongiorno’s model. *Sci. Rep.* **5**, 14640; doi: 10.1038/srep14640 (2015).

## Figures and Tables

**Table 1:** Comparison of values of *f*′′(0) and *g*′(0) with Wang^4^ for the stretching sheet (*λ* > 0).

λ	*f*′′(0)	*g*′(0)	*f*′′(0)	*g*′(0)
	Wang^4^		Present	
0.1	1.14656	−0.86345	1.146561	−0.863452
0.2	1.05113	−0.91330	1.051130	−0.913303
0.5	0.71330	−1.05239	0.713295	−1.051458
1	0	−1.25331	0	−1.253314
5	−10.26475	−2.33810	−10.264749	−2.338099

**Table 2:** Comparison of values of *f*′′(0) and *g*′(0) with Wang^4^ for the shrinking sheet (*λ* < 0).

λ	*f*′′(0)	*g*′(0)	*f*′′(0)	*g*′(0)
	Wang^4^		Present	
−0.5	1.49567	−0.50145	1.495670	−0.501448
−0.75	1.48930	−0.29376	1.489298	−0.293763
−1	1.32882 [0]	0 [∞]	1.328817 [0]	−0.697566 × 10^−7^ [∞]
−1.15	1.08223 [0.116702]	0.297995 [0.276345]	1.082231 [0.116702]	0.297995 [2.763446]

Results in square brackets [ ] are the second (dual) solutions.

**Table 3:** Values of *f*′′(0), *g*′(0), −*θ*′(0) and −*ϕ*′(0) for some values of Brownian motion parameter, *Nb*, thermophoresis parameter, *Nt* and Lewis number, *Le* when Pr = 6.8 for the stretching sheet (*λ* = 0.5).

*s*	*Nb*	*Nt*	*Le*	*f*′′(0)	*g*′(0)	−*θ*′(0)	−*ϕ*′(0)
−0.2 (injection)	0.1	0.3	2	0.659236	−0.937680	0.371027	1.115163
			5			0.307596	1.581147
	0.3	0.1	2			0.313176	0.773216
			5			0.230721	0.978769
	0.1	0.1	2			0.572672	0.701860
			5			0.506525	1.021574
	0.5	0.5	2			0.061587	1.004895
			5			0.034980	1.159491
0 (impermeable)	0.1	0.3	2	0.713294	−1.051458	0.746719	0.366641
			5			0.601097	1.535743
	0.3	0.1	2			0.623690	0.922938
			5			0.436227	1.504253
	0.1	0.1	2			1.092305	0.458058
			5			0.948273	1.220179
	0.5	0.5	2			0.137458	1.221615
			5			0.070106	1.786368
0.2 (suction)	0.1	0.3	2	0.770622	−1.173239	1.275701	0.917460
			5			1.003824	1.085440
	0.3	0.1	2			1.056465	1.049753
			5			0.708499	2.106237
	0.1	0.1	2			1.782383	0.035104
			5			1.524812	1.328678
	0.5	0.5	2			0.259536	1.399831
			5			0.121192	2.481757

**Table 4:** Values of *f*′′(0), *g*′(0), −*θ*′(0) and −*ϕ*′(0) for some values of Brownian motion parameter, *Nb*, thermophoresis parameter, *Nt* and Lewis number, *Le* when Pr = 6.8 for the shrinking sheet (*λ* = −0.5).

*s*	*Nb*	*Nt*	*Le*	*f*′′(0)	*g*′(0)	−*θ*′(0)	−*ϕ*′(0)
−0.2 (injection)	0.1	0.3	2	1.319064	−0.368584	0.036998	0.999060
			5			0.034668	0.660451
	0.3	0.1	2			0.032466	0.367458
			5			0.028427	0.230555
	0.1	0.1	2			0.067026	0.532830
			5			0.063681	0.364182
	0.5	0.5	2			0.004638	0.469622
			5			0.003789	0.267667
0 (impermeable)	0.1	0.3	2	1.495670	−0.501448	0.193542	1.086218
			5			0.167780	1.261482
	0.3	0.1	2			0.166706	0.567484
			5			0.131986	0.641266
	0.1	0.1	2			0.323735	0.599468
			5			0.294383	0.730718
	0.5	0.5	2			0.026919	0.752927
			5			0.017904	0.780476
0.2 (suction)	0.1	0.3	2	1.684907	−0.645128	0.598881	0.280269
			5			0.493117	1.209170
	0.3	0.1	2			0.505697	0.731984
			5			0.365048	1.236568
	0.1	0.1	2			0.911201	0.303766
			5			0.801736	0.917575
	0.5	0.5	2			0.097366	1.018962
			5			0.053513	1.529908

**Table 5:** The critical values of λ.

*λ*_*a*_	*λ*_*b*_	*λ*_*c*_
−1.3455	−1.2465	−1.1629
